# Can Benchmarking Increase the Accuracy of Predicting Biodegradation Rates across Aquatic Ecosystems?

**DOI:** 10.1021/acs.est.6c00470

**Published:** 2026-03-10

**Authors:** Run Tian, Lily M. Weir, Malte Posselt, Kathrin Fenner, Michael S. McLachlan

**Affiliations:** † Department of Environmental Science (ACES), 7675 Stockholm University, 10691 Stockholm, Sweden; ‡ Queensland Alliance for Environmental Health Sciences (QAEHS), 534457 The University of Queensland, 20 Cornwall Street, Woolloongabba 4102, QLD, Australia; § Eawag, Swiss Federal Institute of Aquatic Science and Technology, 8600 Dübendorf, Switzerland; ∥ Department of Chemistry, 30964 University of Zürich, 8057 Zürich, Switzerland

**Keywords:** biodegradation rates, benchmark, chemical grouping, spatiotemporal variability, aquatic ecosystems

## Abstract

Describing and dealing with the large temporal and spatial variability in biodegradation rate constants is a requirement for robust persistence and exposure assessment. Chemical benchmarking uses a well-characterized reference chemical in a manner analogous to an internal standard in analytical chemistry; its measured biodegradation rate constant captures environmental-specific information that is used to predict the variability of the rate constants of other chemicals. We compiled 1656 biodegradation rate constants for 97 chemicals in European and Australian aquatic ecosystems, all measured with the same modified OECD 309 test protocol. Two benchmarking approaches were assessed for their ability to reduce the spatiotemporal variability in the data: (i) normalizing all chemicals to a single benchmark chemical (universal benchmarking); and (ii) grouping chemicals and normalizing the chemicals within each group to a benchmark chemical chosen from within that group (group-specific benchmarking). Universal benchmarking did not reduce the measured variability, while group-specific benchmarking did when the grouping of chemicals was optimized using the data. However, when chemical grouping was predicted based on the molecular fingerprint MACCS (Molecular ACCess System) or initial biotransformation rules, there was no reduction in variability for most chemicals. Group-specific benchmarking has promise as a tool to predict spatiotemporal variability in biodegradation rate constants when an appropriate chemical grouping is possible, but our current understanding of the key chemical features associated with biodegradability is insufficient to reliably group chemicals a priori.

## Graphical abstract

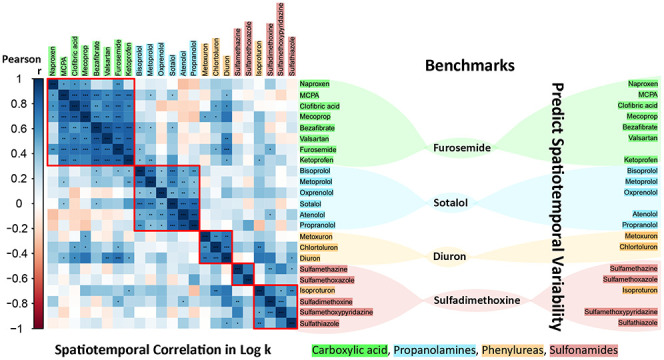

## Introduction

Persistence and exposure assessment require an understanding of the rate of chemical elimination from the environment by degradation.^1–3^ The most important degradation process for many organic chemicals is biodegradation,^4^ and the assessment of biodegradation has a prominent standing in many chemical regulatory frameworks.^1,2,5^ It plays an important role in persistence assessment; for instance, under the European regulation REACH, the OECD 309 guideline for simulation testing of biodegradation in surface water is the recommended test for persistence assessment. Biodegradation is also important in exposure assessment, as illustrated in Arnot et al., who showed that the biodegradation rate was the second largest source of uncertainty (after emissions rate) in an exposure assessment of >12,000 organic chemicals.^6^ However, biodegradation rates are highly variable in the environment, both spatially and seasonally, and this presents challenges for both persistence assessment and exposure assessment.^7–9^ For instance, in assessing persistence against a half-life threshold using a simulation test, how do we choose the environment that is to be simulated, and how do we compare results from tests simulating different environments? In assessing exposure, how do we account for the spatial and temporal variability in biodegradation rates? In this work, we explore one strategy to address these challenges: chemical benchmarking. Note that we approach biodegradation from the perspective of chemical loss, not chemical formation, although the latter can also contribute to the adverse effects of chemicals.

In the natural environment, biodegradation is governed by a complex interplay of several factors. Some of these factors have a direct impact on the biodegradation rate and can be readily quantified. For instance, sorption of the chemical lowers the biodegradation rate, and this can be readily corrected for by normalizing the biodegradation rate to the freely dissolved concentration of the chemical.^10^ Also, pH can influence biodegradation rates of ionizing substances by changing the concentration of the typically more bioavailable neutral form, and this effect can be approximately corrected for by normalizing the biodegradation rate to the concentration of the neutral form of the chemical.^11^ On the other hand, the relationship between other environmental factors and biodegradation rate is more complex and challenging to quantify. For instance, although thermodynamic considerations suggest that temperature should be positively correlated with biodegradation rate, such a correlation is frequently not observed, presumably because temperature also affects the composition of the microbial community.^7,12^ Other environmental variables that can be expected to indirectly affect the biodegradation rate by influencing the composition, functions, and activity of the microbial community include dissolved oxygen concentration, total organic carbon (TOC), and the particle size distribution of sediment, but these variables alone have been shown to have little or no ability to explain the spatial and temporal variability in biodegradation rates.^8,9^ As an alternative to unraveling the complex nature of the links between environmental variables and biodegradation rate, there is an ongoing effort to relate biodegradation rates to taxonomic and functional characteristics of microbial communities. Progress in this area has been constrained by the challenges in identifying the keystone descriptors of the biodegradation capacity of microbial communities from the enormous list of candidate descriptors.^13^ Variability in biodegradation rate can also be examined by studying the difference between chemicals. An important question in this context is to what extent descriptors that have been found to describe the variability in the biodegradation rate of one chemical can be applied to describe the biodegradation rate of other chemicals. In a laboratory study with WWTP inocula, Achermann et al. found that the between-experiment variability in biodegradation rates differed between chemicals, but they observed similar variability for some chemicals expected to undergo similar initial transformation reactions.^14^ Nevertheless, we still lack generally applicable descriptors and algorithms that reliably predict and explain the spatial and temporal variability in biodegradation rates.

Chemical benchmarking is an alternative approach to describing variability.^15^ The concept can be likened to the use of internal standards in chemical analysis, where the change in the signal of a known chemical from the start to the end of the analytical procedure is used to quantify sample-specific losses of chemical and changes in detector sensitivity, which are then employed for the quantification of the chemical of interest. Applied to biodegradation, benchmarking uses a well-characterized chemical (i.e., the benchmark chemical) to quantify environment-specific differences in biodegradation capacity, which are then employed for the quantification of the environmental variability of the biodegradation rate constant of the chemical of interest. This approach assumes that the relative reactivity of a group of chemicals is approximately constant in space and time, for which some support is provided by the work of Achermann et al.^14^ When this assumption is valid, then the biodegradation rate of a benchmark chemical from the group can be used as a proxy for the variable biodegradation behavior of other chemicals in the group.^16–19^ In other words, by measuring the biodegradation rate constant of the benchmark chemical in the system of interest, the rate constants for other chemicals can be estimated, provided that their relative biodegradation rate constants in a reference system are known. This would substantially reduce experimental effort for accurate persistence and exposure assessment. In exposure assessment, the biodegradation rate of benchmark chemicals could be measured across multiple systems of interest. The resulting benchmark-specific rate constants could then be used to scale the biodegradation rates of additional test chemicals that have been measured in a reference experiment alongside the benchmark, providing a map of test chemical variability from just a single experiment. In persistence assessment, benchmark chemicals could be included in simulation tests across diverse systems, and the rate constants of the benchmark chemical could be used to correct for the variability in biodegradation rate of the test chemical across systems, helping to describe the variability and increase confidence in persistence estimates.

Exploration of the benchmarking approach requires a sufficiently large data set of consistently generated biodegradation rate constants. However, existing studies are generally based on biodegradation tests with limited environmental relevance, and they typically focus on isolated factors (e.g., temperature, salinity) or specific environmental conditions,^20^ resulting in fragmented and poorly comparable data sets. Recently, we modified the OECD 309 guideline for simulating biodegradation of chemicals in surface water, a standardized test widely used for persistence assessment in chemical regulation, so that it would give more environmentally relevant measures of biodegradation rates.^21^ This laboratory test incubates surface water that has been inoculated with the test chemical, and derives a first-order rate constant *k* from the attenuation of the test chemical over time. With the modified guideline, we conducted a series of experiments to measure the biodegradation of 97 chemicals in samples from 38 different locations in European rivers and Australian surface water bodies.^7–9^ At four locations, experiments were also conducted during different seasons.^7^ The resulting methodically coherent data set of 2265 first-order rate constants provides a unique opportunity to study the spatial and temporal (seasonal) variability of biodegradation and explore the benchmarking approach.

In this study, we use this data set to evaluate the utility of chemical benchmarking. We assessed the predictive potential of benchmarking by assessing how well it can describe the variability in these data. Two different benchmarking strategies were applied: universal benchmarking (UBM) and group-specific benchmarking (GBM). UBM uses one benchmark chemical to predict the variability of all other chemicals. It tests the hypothesis that all microbial communities are functionally equivalent across systems for the biodegradation of various chemicals, such that the behavior of one chemical can serve as a proxy for all others. To implement UBM, each chemical was tested as a benchmark against the remaining chemicals, with the chemical selected as the best universal benchmark being the one that most strongly reduced the variability in rate constants across different locations. For GBM, a given benchmark chemical is only used for a specific group of chemicals. It tests the hypothesis that functional differences among microbial communities are reflected in groups of structurally or biodegradation-mechanism related chemicals exhibiting similar biodegradation patterns across systems. GBM was implemented using three data-independent methods and one data-dependent (i.e., optimized) method to group the chemicals according to expected similarity in biodegradation behavior, before selecting the benchmark chemical for each group in a similar manner as for UBM. In order to provide context for the efficacy of the benchmarking strategies, we included normalization to TOC as an alternative method for describing spatial variation. The efficacy of the different strategies was assessed using the change in the variance of the log *k* for each chemical, the expectation being that application of benchmarking (or, in the case of TOC, normalization) would markedly reduce the variance for the majority of the chemicals.

## Materials and Methods

### Biodegradation Rate Constant Data Set

The experimental data for biodegradation rate constants (*k*, d^–1^) were collected in our previous studies for 38 different aquatic ecosystems in Europe (31 ecosystems) and Australia (7 ecosystems). In our experiments, biodegradation was distinguished from other degradation processes using abiotic controls. Our modifications to the guideline included: (i) calculating the rate constant using only the initial attenuation data, whereby we assumed that a departure from the initial first order attenuation behavior signaled a departure of the microbial community in the incubation flask from its initial (environmental) state; (ii) adding surface sediment to the incubation (50 g L^–1^), which was done because the initial biodegradation rate was negligible (<LOQ) in sediment-free incubations; (iii) spiking low concentrations of test chemicals (1 μg L^–1^) to reduce impacts of the test chemical on the microbial community; (iv) spiking multiple chemicals simultaneously, after it was found that this did not impact the test result.^21^ The 38 experiments were conducted between 17 March 2022 and 28 April 2023. The sampled microbial habitats were diverse, including both pristine and contaminated systems, and both marine and freshwater systems. To account for the known and quantifiable influence of sorption on apparent *k* values and thereby reduce the number of variables influencing the biodegradation rate, *k* was corrected by normalizing to the fraction of the chemical in the dissolved form.^7–9^

Only valid *k* values (i.e., significantly different from zero) were included in our analysis.^7–9^ This resulted in a total of 2265 *k* values for 97 chemicals. To avoid introducing large biases, outliers were identified by the interquartile range (IQR) method and removed,^22^ which reduced the data set to 2042 *k* values for 97 chemicals. Since we were studying spatial variability, only chemicals that had *k* values in at least half of the ecosystems were considered further. This final test data set contained 1663 *k* values for 62 chemicals (see the Supporting Data set).

A second data set was also employed in which *k* was additionally corrected to account for differences in pH between the experiments (*k*_pH7_, d^–1^). Apparent *k* values were corrected to a reference pH of 7 using the ratio of the neutral fraction of the test chemical at pH 7 and at the pH in the experiment.^7–9^ Similar to the correction for the dissolved fraction, this correction was also applied to reduce variability arising from readily quantified factors, thereby allowing the subsequent benchmarking testing to focus on residual variability that was more likely linked to differences in microbial community composition and function. After removing outliers and chemicals that had *k*_pH7_ in less than half of the ecosystems, there were 1656 *k*_pH7_ values for 62 chemicals. The data are provided in the Supporting Data set. Note that all of the assumptions underlying the correction for differences in pH might not be completely satisfied in all cases, and that the procedure has been shown to overcorrect to some extent.^11^

In each experiment used to determine the *k* values, the test chemicals were spiked as a mixture, and the estimate of *k* for each individual chemical was based on all the data from three test treatment replicates.^7–9^ Each *k* value was derived from an independent experiment conducted with water and sediment sampled at a specific location and sampling time. At the low chemical concentrations spiked, the estimate of *k* is not influenced by the use of a mixture.^21,23^ The standard deviations of log *k* between the three test treatment replicates were collected for each experiment from our previous papers and used as a measure of experimental uncertainty.^7–9^

### Benchmarking

The fundamental assumption of chemical benchmarking, namely that the ratio of the property of the chemical of interest and the benchmark is constant in space and time, is expressed by

1 $$\frac{k_{i,j}}{k_{\mathrm{BM},j}}=\frac{k_{i,\mathrm{ref}}}{k_{\mathrm{BM},\mathrm{ref}}}$$

where *k_i,j_* is the property (here biodegradation rate constant) of chemical *i* at site *j*, BM indicates the benchmark chemical, and ref indicates the reference site (i.e., reference experiment at a certain site). In applying benchmarking, one measures the ratio of *k* for the chemical of interest and the benchmark chemical at a reference site (*k*_*i*,ref_/*k*_BM,ref_, thereby characterizing the chemical properties of *i* affecting *k*) as well as *k* for the benchmark chemical at site *j* (*k*_BM,*j*_, thereby characterizing the site properties (with respect to the reference site) affecting *k*). *k*_*i*,*j*_ can then be calculated as the remaining unknown in eq 1.

The utility of benchmarking for predicting the spatial and temporal variability in biodegradation rate constants was evaluated by assessing whether applying it reduces the observed variability in our data set. We assessed the efficacy of benchmarking by first selecting a benchmark chemical and then calculating *k*_*i*,*j*_/*k*_BM,*j*_ (or *k*_*i,j*_^′^, where the symbol ′ signifies benchmarked) for the complete data set. In the case of a good benchmarking result, *k*_i,j_/*k*_BM,j_ for a given chemical *i* would be constant across all sites *j* (and equal to *k*_i,ref_/*k*_BM,ref_). Our quantitative measure of efficacy was the reduction in the spatiotemporal variability for each chemical between the original data set and the benchmarked data set. The spatiotemporal variability was defined as the standard deviation (stddev) of log *k* and log *k*′, respectively. The efficacy in reducing spatiotemporal variability after performing benchmarking (REM) was quantified by the change in the stddev of log *k*, a reduction being a positive benchmarking outcome:

2 $${\mathrm{REM}}_{i}={\mathrm{stddev}}_{i}-{\mathrm{stddev}}_{i}^{\prime }$$

3 $${\mathrm{stddev}}_{i}=\sqrt{\frac{1}{\left(N-1\right)}\sum_{j=1}^{N}{\left(\log k_{i,j}-\overset{®}{\log k_{i}}\right)}^{2}}$$

4 $${\mathrm{stddev}}_{i}^{\prime }=\sqrt{\frac{1}{\left(N-1\right)}\sum_{j=1}^{N}{\left(\log k_{i,j}^{\prime }-\overset{®}{{\log k}_{i}^{\prime }}\right)}^{2}}$$

where *N* is the number of sites that had valid rate constants for *i*. When the rate constant of the selected benchmark chemical was not valid at a given site, the residual from the log *k* (i.e., $\log k_{i,j}-\overset{®}{\log k_{i}}$ ) was used to fill the missing residual of log *k*′ (i.e., $\log k_{i,j}^{\prime }-\overset{®}{{\log k}_{i}^{\prime }}$ ) in the calculation of stddev’ and REM.

Two benchmarking approaches were tested:

1. Benchmark the rate constants of all chemicals against one potential benchmark chemical (universal benchmarking, UBM);
2. Group the chemicals and identify a different benchmark chemical for each group (group-specific benchmarking, GBM).

Each chemical was tested as a benchmark against the remaining chemicals, and the chemical that provided the largest median REM was selected as the best benchmark. For UBM, this was performed for all chemicals to find the best universal benchmark, while for GBM, this was performed for the chemicals within the group to find the best benchmark for the group. This selection procedure ensured that the pattern and the magnitude of the variability in the biodegradation rate constant for the benchmark chemical were similar to those for the test chemicals.

To provide context for the efficacy of the benchmarking approaches, we included normalization to TOC as an alternative method for reducing spatial variation. The efficacy of TOC normalization was also quantified by the change in stddev before and after the normalization.

### Chemical Grouping Approaches for GBMs

In order for GBM to be a useful predictive tool, it is necessary to be able to predict which group the chemical of interest belongs to. To guide the selection of groups for GBM, we performed agglomerative hierarchical clustering to group chemicals according to the similarity of their biodegradability-related characteristics/descriptors.^24^ The dendrogram function from the Cluster package (v.2.1.6)^25^ in R was used to perform clustering. The Euclidean distance between two diffraction profiles was used as the similarity metric, and Ward linkage was used to measure the cluster dissimilarity.

The chemicals were grouped based on two approaches: (1) Data-dependent. Chemicals were grouped based on how well their log *k* values correlated with each other. For the hierarchical clustering, each chemical i was represented by a vector consisting of the Pearson coefficients for the correlation of log *k* of chemical i against log *k* of each of the other chemicals. (2) Data-independent. Chemicals were grouped based on the similarity of their structures. Three different descriptors of chemical structure were tested: Molecular ACCess System (MACCS) molecular fingerprints, predicted initial biotransformation reactions (btrules), and predicted probabilities for each btrule (btrules_prob). To transform the raw chemical structures into digitally readable information, each chemical was represented as a vector consisting of a set of molecular fingerprints or biotransformation rules that were encoded into a binary bit string. MACCS grouped chemicals by chemical functional groups, and each chemical was represented as a vector consisting of 166 MACCS descriptors.^26^ btrules and btrules_prob grouped chemicals according to the likelihood of specific types of biotransformation reactions. They were collected for each of the 97 test chemicals from enviPath.^27^ Each chemical was represented as a vector consisting of 208 btrule descriptors (or 65 btrules_prob descriptors) that were encoded into a binary bit string. There were fewer btrules_prob descriptors because the predicted probability for 143 btrules was zero for all test chemicals. The MACCS, btrules, and btrules_prob were all downloaded from a published workflow (https://github.com/FennerLabs/pepper). More details on the test chemicals and the three sets of descriptors can be found in the Supporting Data set.

We used the chemical clusters from the hierarchical clustering of the Pearson correlation coefficient to generate a case of optimized GBM, since this clustering grouped the chemicals according to the similarity in their spatiotemporal variability. The outcomes of GBM based on MACCS, btrules, and btrules_prob were compared to outcomes of the optimized GBM to explore whether an a priori, data-independent approach can provide comparable results to an optimized GBM. The clustering for the optimized GBM ensured that there were at least two chemicals in each cluster. The number of groups obtained from this clustering was used for the clustering based on MACCS, btrules, and btrules_prob. The chemicals were further clustered into a larger number of groups to explore whether the efficacy of GBM could be improved by increasing the similarity of the chemicals in each group.

## Results and Discussion

### Descriptive Statistics of Biodegradation Rates

The values of *k* and *k*_pH7_ both varied from 0.001 to 29.1 d^–1^ (Supporting Data set). The median *k* was 0.14 (half-life ∼ 5 days). The log *k* and log *k*_pH7_ of all chemicals were significantly different between the 38 ecosystems (ANOVA, *P* < 0.01) with stddevs ranging from 0.18 to 0.82, and 0.18 to 0.91, respectively (Figure S1). The median stddevs of log *k* and log *k*_pH7_ between all ecosystems were similar at about 0.4, corresponding to a fold difference of factor 2.5 (Figure S1). The stddev of log *k* between the three test treatment replicates was used to quantify the uncertainty in the measurements. The median for all experiments was 0.10, which was ∼ 4 times lower than the median stddev of log *k* between the 38 ecosystems (Figure 1). Therefore, the observed spatiotemporal variability was not strongly influenced by the measurement error.

**Figure 1:**
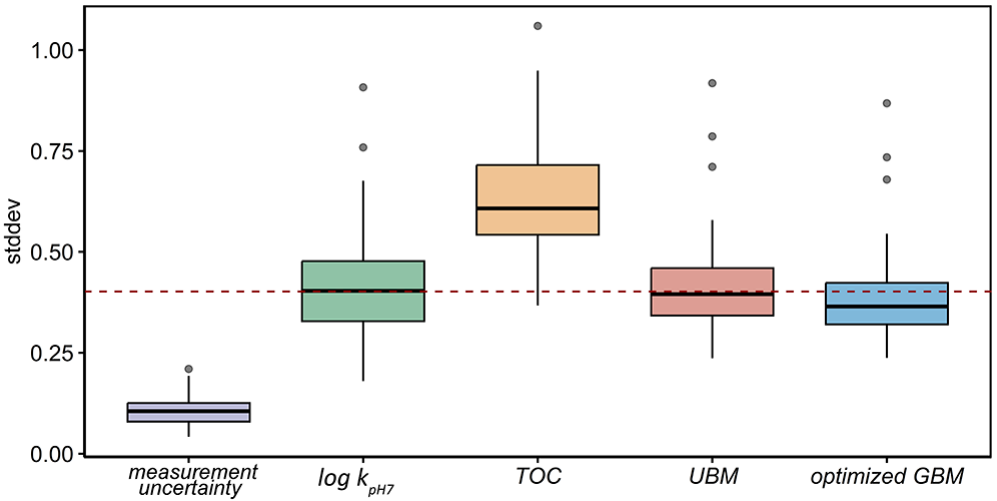
Boxplot showing the standard deviation (stddev) of log *k*_pH7_ (d^–1^) between three test treatment replicates (measurement uncertainty), the stddev of log *k*_pH7_ between all aquatic ecosystems (log *k*_pH7_), and the stddev of log *k*_pH7_ after conducting total organic carbon normalization (TOC), UBM using the best-performing benchmark (UBM), and the optimized GBM using the best-performing benchmark in each chemical group (optimized GBM). The boxes show the distribution (including the median, 25th and 75th percentiles, minimum, and maximum) of the stddev. The dots represent the outliers.

In addition to the spatiotemporal variability being similar for *k* and *k*_pH7_, the efficacy of the different benchmarking strategies was also similar for *k* and *k*_pH7_. Therefore, we present only the results for *k*_pH7_ in the following, while the results for *k* are provided in the Supporting Information (SI).

### Universal Benchmarking (UBM)

To evaluate UBM, each chemical was tested as a benchmark against the remaining chemicals. Only two candidate universal benchmark chemicals (DIU (diuron) and FFA (flufenacet)) reduced the stddev of log *k*_pH7_ for more than 50% of the test chemicals (i.e., had a median REM > 0, Figure S2). However, even though these two chemicals were the best universal benchmarks, they did little to reduce the variability. The median REMs for DIU and FFA were both about 0.0004 (i.e., the median reduction in variability was a factor of 1.02 and 1.0009). We attribute the poor performance of UBM to the diversity in chemical structures and microbial communities. One benchmark chemical alone is insufficient to predict a substantial fraction of the spatiotemporal variability in biodegradation rate constants of structurally diverse chemicals across a wide range of microbial communities.

In contrast to the slight decrease for universal benchmarking, TOC normalization was found to increase the median stddev of log *k*_pH7_ between experimental ecosystems by 0.21 (a factor of 1.62, Figure 1). Even though UBM performed better than TOC normalization, neither of the two candidate universal proxies described the variability in biodegradation rate constants for individual chemicals well. The limited ability of UBM and TOC normalization to reduce variability indicates that microbial communities cannot be assumed to be functionally equivalent across systems. The relative capacities of microbial communities to degrade different chemicals vary across space and time.

### Optimized Group-Specific Benchmarking (GBM)

The hierarchical clustering based on the Pearson correlation coefficient yielded eight clusters of chemicals (Figure 2). The variance in spatiotemporal pattern was lower within the clusters, and a different benchmark chemical was identified for each cluster. Compared to UBM, the optimized GBM was much more effective at reducing the spatiotemporal variability. The stddev was reduced for 87% of chemicals (excluding the benchmark chemicals) when using the best-performing benchmark in each group (Figures 1 and 3). The median REM in each group ranged from 0.01 to 0.13 (corresponding to a reduction of stddev by a factor ranging from 1.02 to 1.35, see Figure 2). Subdividing the chemicals further to create more groups (16 groups) somewhat increased the median REM (Figure S4). However, increasing the number of clusters can make it more difficult to find common rules that determine inclusion in a cluster (e.g., chemical structure or initial biotransformation rules).

**Figure 2:**
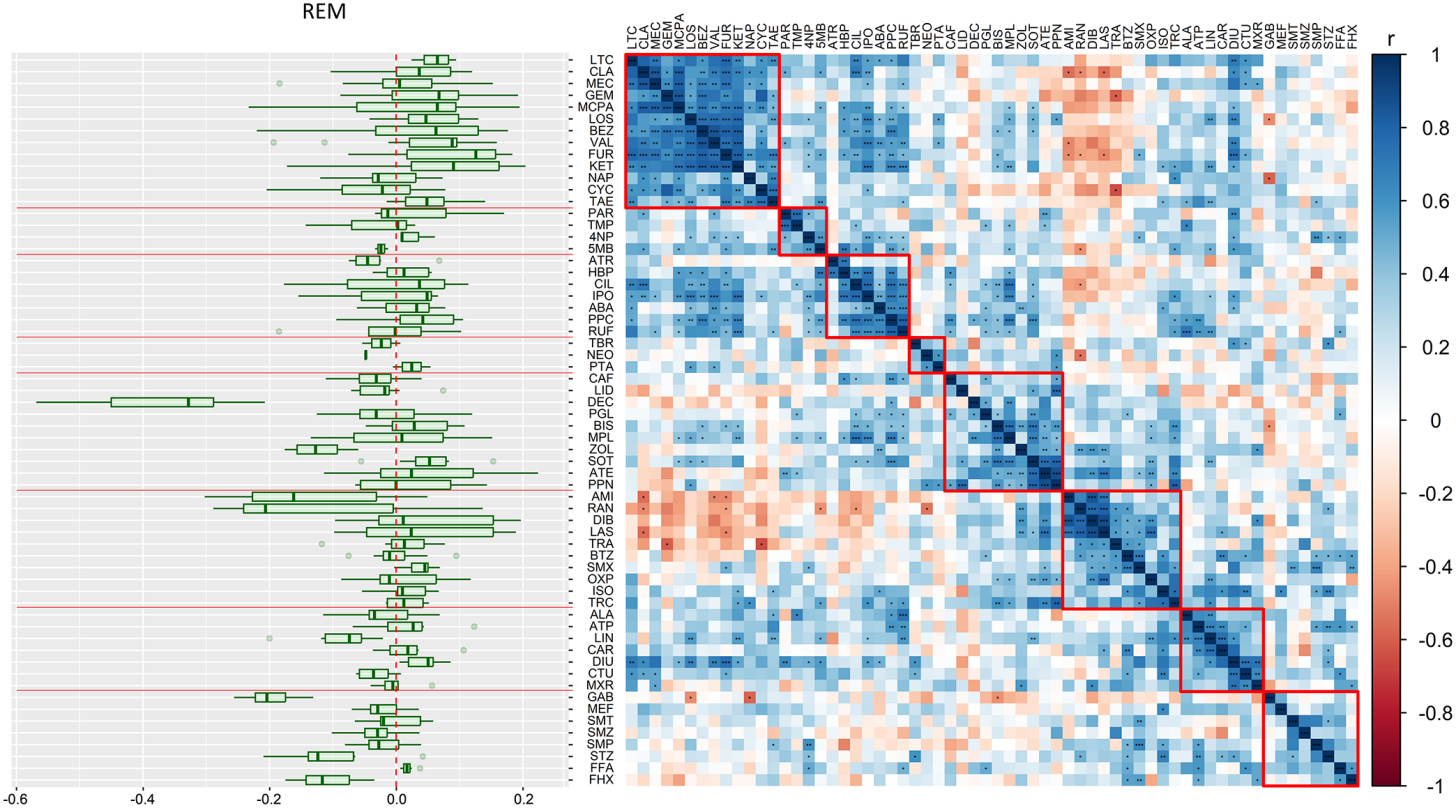
**Right panel**: Hierarchical clustering heatmap based on the magnitude of the correlation coefficient (r, Pearson) of log *k*_pH7_ (d^–1^) between pairs of chemicals. The significance of the correlations (*P*, Pearson) is denoted by * (*P* < 0.05), ** (*P* < 0.01), *** (*P* < 0.001). The chemicals were classified into eight clusters (chemical groups). In each cluster, the rate constants of all chemicals were benchmarked against one potential benchmark chemical. **Left panel**: The boxplot shows the median, 25th and 75th percentiles, minimum, and maximum (calculated by the Fivenum function in boxplot (ggplot2, v.3.5.1) in R) of the magnitude of the reduction efficacy (REM) for each potential benchmark, i.e., the change in the stddev of log *k*_pH7_ across ecosystems when it was used as the benchmark for other chemicals in the same cluster. Positive values of REM mean positive benchmark results. Red lines show the boundaries of the chemical groups. For elaboration of the chemical name abbreviations see the Supporting Data set.

**Figure 3:**
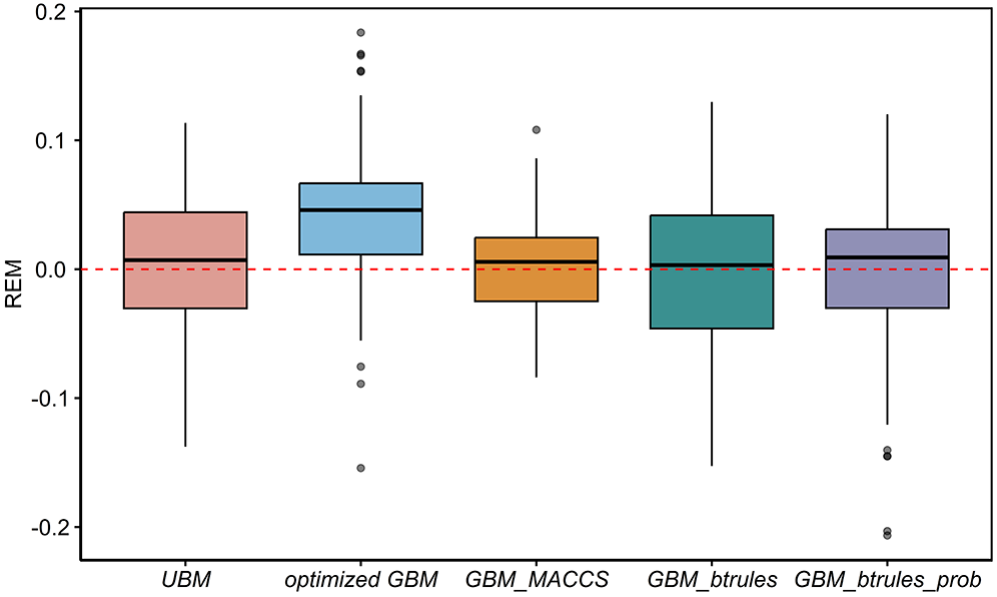
Efficacy of universal benchmarking (UBM) and group-specific benchmarking (GBM) using chemical groups identified based on the Pearson correlation coefficients derived from the data set (optimized GBM) and on different descriptors (MACCS, btrules, and btrules_prob). The boxplot shows the median, 25th and 75th percentiles, minimum, and maximum of the magnitude of the reduction efficacy (REM), i.e., the change in the stddev of log *k*_pH7_ (d^–1^) across systems when using the best-performing benchmark among all tested chemicals for UBM and the best-performing benchmark in each group for GBM. A positive REM was a positive benchmark result.

### Data-Independent GBM

To explore if the performance of the optimized GBM could be approached by a data-independent GBM, we used three kinds of chemical descriptors to group the chemicals, i.e., one that describes molecular structure (MACCS) and two that describe the predicted likelihood of specific types of initial biotransformation reactions (btrules and btrules_prob). These methods were compared with the optimized GBM according to (a) the composition of the chemical groups (Figure 4); and (b) REM (Figure 3). To facilitate parallel comparison, the chemicals were always clustered into eight chemical groups using Ward’s method (Figure S5).

**Figure 4:**
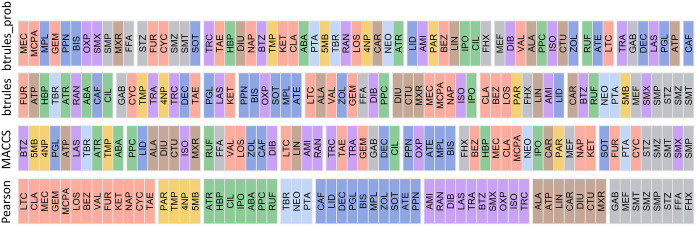
Chemical groups (split) identified by the clustering of chemicals based on Pearson correlations of log *k*_pH7_, on MACCS, on btrules, and on btrules_prob. Within each group, chemicals were similar in terms of the respective features (i.e., Pearson correlations of log kpH7, MACCS, btrules, and btrules_prob). The color marks chemicals belonging to the same group based on the Pearson correlations. For elaboration of the chemical name abbreviations see Supporting Data set.

None of the three grouping methods produced groups that were similar to those obtained from the optimized GBM (Figure 4). This suggests that it is not trivial to find a consistent grouping of chemicals based on different chemical descriptors/features. Only three chemical classes were grouped consistently using both the data-dependent and data-independent methods. Propanolamines (ATE, BIS, MPL, OXP, PPN, SOT), phenylureas (CTU, DIU, ISO, MXR), and sulfonamides (SMP, SMT, SMX, SMZ, STZ) usually clustered closely according to either MACCS or btrules, as well as using optimized GBM (Figures 2 and 4). Similarities in seasonal and/or spatial variability within these chemical groups were also observed in our previous studies.^7,8^ When we further divided the chemicals into more clusters based on MACCS, btrules, and btrules prob (16 clusters, Figure S5), most of the propanolamines, phenylureas, and sulfonamides were still clustered together. This suggested that both the molecular structures and the predicted initial biotransformation rules for chemicals within these three groups were more similar compared to the other chemicals studied. The observation that other groups were separated as the number of clusters increased suggests lesser similarity in molecular structure and predicted initial biotransformation rules within those groups. The results show that using different chemical descriptors strongly affects the chemical grouping outcomes. Further work is needed to identify descriptors that differentiate the key chemical features governing the biodegradability of a chemical.

Additionally, we found some chemical groups for which features or factors not captured in MACCS, btrules, or btrules_prob were important determinants of the spatiotemporal pattern of biodegradation rate constants. For instance, we found a group of chemicals for which log *k*_pH7_ was highly correlated, yet did not cluster closely according to MACCS, btrules, or btrules_prob (Figure 4, chemicals colored in red). Most of the chemicals in this group are carboxylic acids (BEZ, CLA, FUR, KET, MCPA, MEC, NAP, and VAL).

Despite the agreement for a few chemical classes in terms of data-dependent and data-independent clustering, all three of the data-independent GBM methods performed poorly with regard to reducing the spatiotemporal variability in the data set (Figure 3). The median REM using all 3 methods was about 0.005 (a factor of 1.01), which was much lower than the median REM of the optimized GBM (0.046, a factor of 1.11). All three methods only reduced the variability for about 50% of the chemicals (Figure 3). However, for the three chemical groups that always clustered closely using different methods (propanolamines, phenylureas, and sulfonamides), all the methods led to an improvement in reducing the variability compared to the best UBM (Figures S2, S6). For the remaining majority of tested chemicals, however, the results indicate that simply grouping based on either chemical structures or initial biotransformation rules could not increase the accuracy of predicting biodegradation in space and time.

Btrules emphasize “transformation-relevant functional groups”. A functional group triggering a btrule can be either a biotransformation promoter or inhibitor. This information is conveyed to some extent by the btrules_prob. A given chemical usually has several btrules triggered by different functional groups. The complex interplay between functional groups probably contributed to the deviation of the clustering of chemicals based on btrules from the optimized GBM. Contrary to expectations, the complexity was not captured better by using rule probabilities rather than just the triggering of rules. MACCS describes the chemical structure but lacks the identification of rate-determining substructures and prioritization of the functional substructures for biodegradation. Overall, the outcomes of GBM based on chemical descriptors demonstrate that a better understanding of the key chemical features governing biodegradation is required for GBM to become a viable tool for describing the spatiotemporal variability in biodegradation.

### Implications and Perspectives

In this study, we tested the efficacy of different benchmarking methods in predicting the spatiotemporal variability of biodegradation rate constants in aquatic ecosystems using two data sets of 1663 *k* and 1656 *k*_pH7_ values. We found that a universal proxy (TOC or a single chemical benchmark) could not predict the spatiotemporal variability. Benchmarking within chemical groups that exhibit a strong spatial and temporal correlation of biodegradation rate constants (optimized GBM) increased the efficacy of benchmarking for predicting the variability. The grouping of chemicals that gave optimized GBM could not be reproduced by grouping the chemicals according to MACCS or predicted initial biotransformation rules, except for three specific groups of chemicals (propanolamines, phenylureas, and sulfonamides). This suggests that the observed biodegradation kinetics were the result of a complex interplay of chemical structure and microbial community functions that cannot be sufficiently captured by the three metrics of chemical similarity tested in this study. At present, no descriptors have yet been found that allow spatiotemporally similar biodegradability groups to be identified a priori. Optimized GBM is a promising method for predicting the variability. However, the need to measure biodegradation rates at diverse sites for a large number of chemicals limits its application to chemicals for which such data are available, which excludes most contaminants in aquatic ecosystems and new chemicals. For future research, expanding data sets across diverse environmental systems and chemical classes will help refine chemical grouping and benchmark selection criteria.

In addition to the prediction of the spatiotemporal variability of environmental biodegradation, this study also contributes to explaining this variability. By applying benchmarking after correcting for known and quantifiable factors (i.e., sorption and pH-dependent speciation), the remaining variability largely reflects differences in microbial community composition and function that are a consequence of multiple environmental factors. UBM effectively tested the hypothesis that all microbial communities have more or less the same combination of functions for chemical biodegradation. Its limited performance refutes this hypothesis, at least across diverse aquatic systems. GBM tested the hypothesis that the combination of biodegradation functions differs among microbial communities, but that a given function can apply to multiple chemicals. This hypothesis is supported by the more consistent biodegradability within certain groups of chemicals and the improved performance of optimized GBM compared to UBM. In this respect, our results are consistent with recent research that showed that microbial communities in similar systems can differ in composition and biodegradation potential over time and between locations,^12,28,29^ and that the variability in biodegradation potential showed similar patterns within several groups of structurally similar chemicals.^14,28,30^ Our study demonstrates that such functional differences among microbial communities across diverse systems are likely to influence the biodegradation rates of large numbers of chemicals. At the same time, the failure of our three data-independent grouping strategies demonstrates that further work is required to identify the factors that explain the biodegradation grouping of organic micropollutants.

The efficacy of benchmarking can be impacted by the quality of the data. In our data set, the spatiotemporal variability (expressed as the stddev of log rate constants) was a factor of 4, on average, larger than the measurement uncertainty. This indicated that the influence of measurement uncertainty on the spatiotemporal variability in our data set was small. However, the missing values (gaps) in the data set of rate constants can also influence the efficacy of benchmarking. Most of the gaps were caused by the removal of invalid data (i.e., the rate constants were not significantly different from zero). When gap-filling data for which the confidence intervals intersect zero and then log-transforming the data, as was done in our previous studies,^7–9^ very large uncertainties can be introduced. In the present study, we therefore excluded all invalid data to reduce the uncertainty. No significant Pearson or Spearman correlation was observed between REM and the number of gaps (*P* > 0.05). However, excluding data excludes information about the biodegradation capacity of certain sites for a specific chemical or chemical group. This can impact the evaluation of spatiotemporal variability and the efficacy of benchmarking. Reduction of this source of uncertainty, for instance, by improving the experimental method to reduce the number of invalid data or by developing better gap-filling algorithms, could improve the evaluation.

This study is a start in exploring the use of benchmarking to predict spatiotemporal variability in biodegradation rates in the environment. The chemical space covered in this study is still limited (the tens of chemicals are merely the tip of the iceberg), which means that the benchmark chemicals identified here cannot be considered universally applicable across all chemical classes and all environmental systems. Nevertheless, the current study demonstrates the potential of GBM in predicting biodegradation rate constants of chemicals across ecosystems. This provides an empirical basis for further exploration of benchmarking strategies and the development of predictive tools that could be applied to a broader chemical space in future studies. More insights can be expected as more high-quality data become available and as we develop our understanding of the chemical structural features and environmental properties that regulate biodegradation. In the longer term, we hope that a better understanding of the factors causing the spatiotemporal variability will facilitate the estimation of biodegradation rate constants that are sufficiently well characterized for persistence and exposure assessments.

## Supplementary Material





## Notes

Test chemicals, chemical descriptors, biodegradation rate constants (XLSX)
Figure S1. Heatmap showing the standard deviation (stddev) of log *k* and log *k*_pH7_ between all aquatic ecosystems. Figure S2. Heatmaps showing the REM of UBM for log *k* and log *k*_pH7_. Figure S3. Hierarchical clustering heatmap (right) based on the magnitude of the Pearson correlation coefficient of log *k*. Figure S4. Hierarchical clustering heatmap (right) based on the magnitude of the Pearson correlation coefficient of log *k*_pH7_. Figure S5. Agglomerative hierarchical clustering of chemicals based on MACCS, btrules, and btrules_prob. Figure S6. Boxplot showing the magnitude of the change in the stddev of log *k*_pH7_ across systems for each chemical when it was used as the benchmark for other chemicals in the same group (PDF)
