# Supplementary material for: Can Benchmarking Increase the Accuracy of Predicting Biodegradation Rates across Aquatic Ecosystems?
Source: Environ Sci Technol. 2026 Mar 10;60(13):10234–41. doi: 10.1021/acs.est.6c00470 (PMC13063413; doi:10.1021/acs.est.6c00470)
Supplement: Supplementary file 2 [file es6c00470_si_002.pdf]

Can benchmarking increase the accuracy of predicting biodegradation rates across aquatic ecosystems?

Run Tian,[1] Lily M. Weir,[2] Malte Posselt,[1] Kathrin Fenner,[3,4] Michael S. McLachlan[1]*

[1]Department of Environmental Science (ACES), Stockholm University, 10691 Stockholm, Sweden

[2]Queensland Alliance for Environmental Health Sciences (QAEHS), The University of Queensland, 20 Cornwall Street, Woolloongabba 4102, QLD, Australia

[3]Eawag, Swiss Federal Institute of Aquatic Science and Technology, 8600 Dübendorf, Switzerland

[4]University of Zürich, Department of Chemistry, 8057 Zürich, Switzerland

*Corresponding author: run.tian@aces.su.se

Number of pages: 7

Number of figures: 6

List of Figures

Figure S1. Heatmap showing the standard deviation (stddev) of log k and log k_{pH7} between all aquatic ecosystems 3

Figure S2. Heatmaps showing the REM of UBM for log k and log k_{pH7} 4

Figure S3. Hierarchical clustering heatmap (right) based on the magnitude of the Pearson correlation coefficient of log k. The chemicals were classified into eight clusters. The boxplot (left) shows the magnitude of the change in the stddev of log k across systems for each chemical when it was used as the benchmark 5

Figure S4. Hierarchical clustering heatmap (right) based on the magnitude of the Pearson correlation coefficient of log k_{pH7}. The chemicals were classified into 16 clusters. The boxplot (left) shows the magnitude of the change in the stddev of log k_{pH7} across systems for each chemical when it was used as the benchmark 5

Figure S5. Agglomerative hierarchical clustering of chemicals based on MACCS, btrules, and btrules_prob. The dendrogram yielded eight clusters and 16 clusters, respectively, for the three descriptors 6

Figure S6. Boxplot showing the magnitude of the change in the stddev of log k_{pH7} across systems for each chemical when it was used as the benchmark for other chemicals in the same group. The grouping of chemicals was based on MACCS, btrules, and btrules_prob, respectively 7

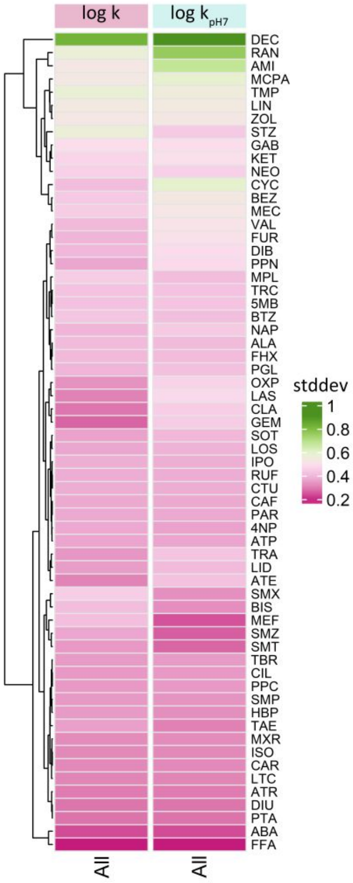

Figure S1. Heatmap showing the standard deviation (stddev) of log k (d^{-1}) and log k_{pH7} (d^{-1}) between all aquatic ecosystems (All). For elaboration of the chemical name abbreviations see Supplemental Dataset.

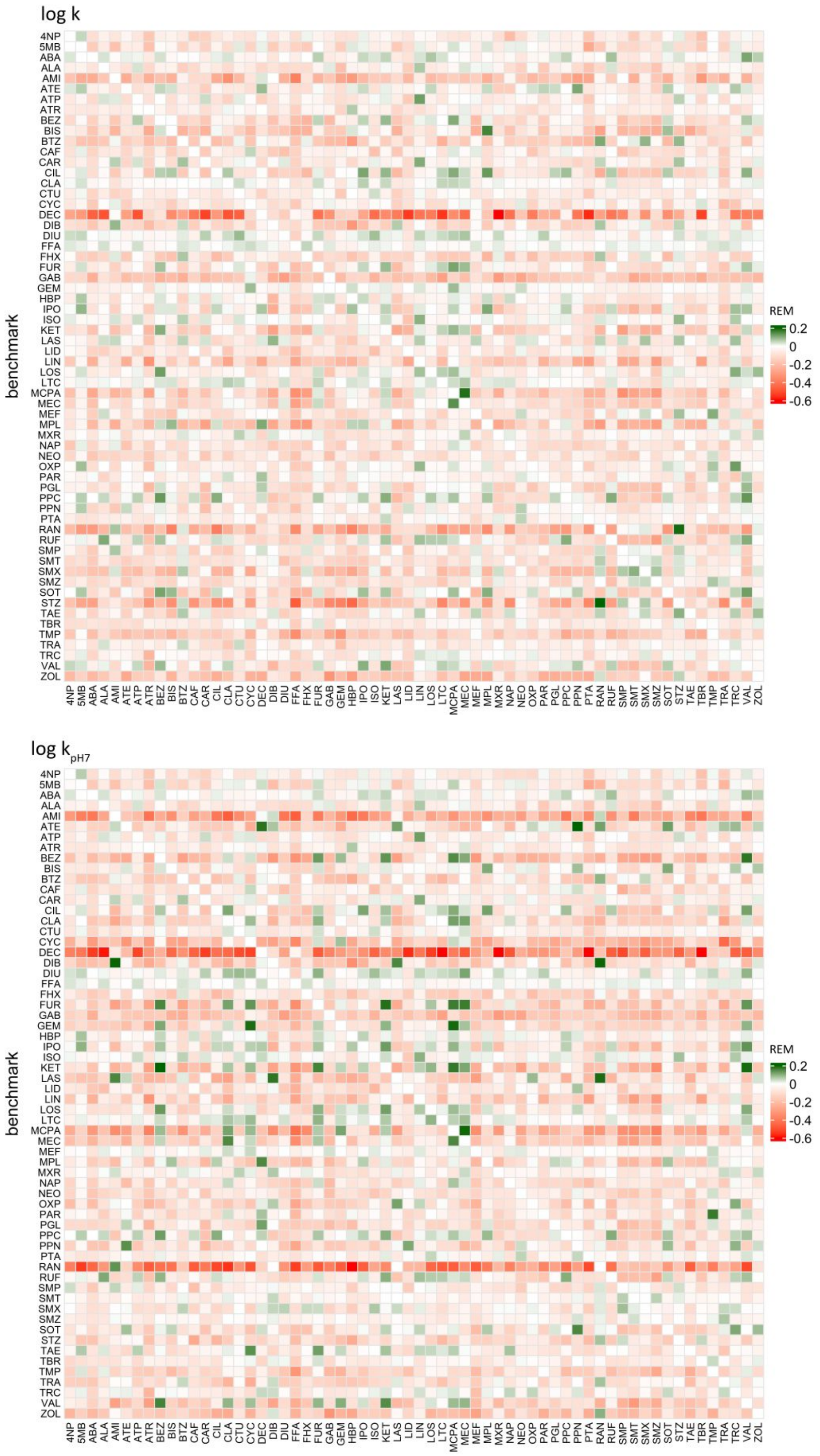

Figure S2. Heatmaps showing the magnitude of reduction efficacy (REM, i.e., the change in the stddev of log k across systems for each chemical (x-axis) when a specific chemical (y-axis) was used as the benchmark) of UBM for log k and log k_{pH7}. A positive REM was a positive benchmark result. For elaboration of the chemical name abbreviations see Supplemental Dataset.

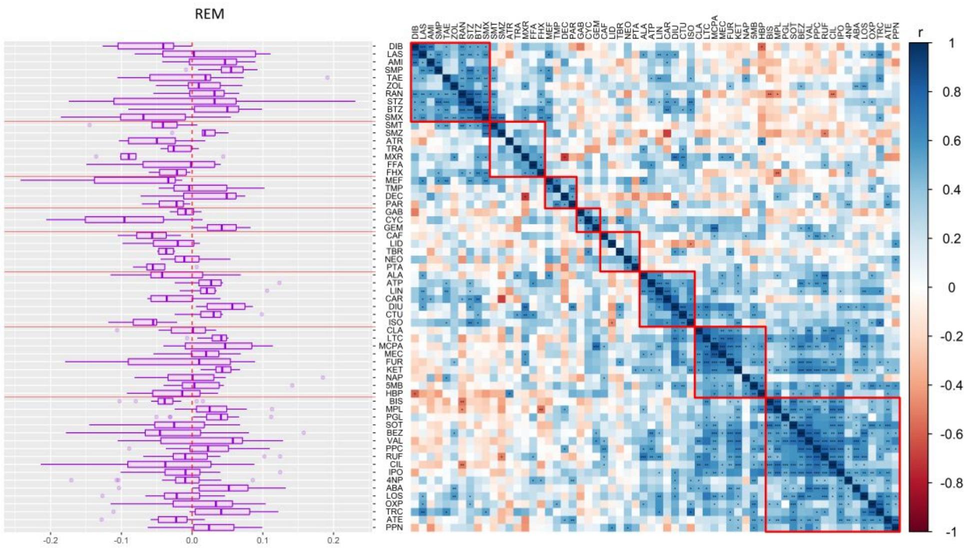

Figure S3. Hierarchical clustering heatmap (right) based on the magnitude of the Pearson correlation coefficient (r) of log k (d^{-1}). The significance of the correlations (P) is denoted by * ($P < 0.05$), ** ($P < 0.01$), *** ($P < 0.001$). The chemicals were classified into eight clusters (chemical groups). In each group, the rate constants of all chemicals were benchmarked against one potential benchmark chemical. The boxplot (left) shows the magnitude of the reduction efficacy (REM), i.e., the change in the stddev of log k across systems for each chemical when it was used as the benchmark. A positive REM was a positive benchmark result. Red lines show the boundaries of the chemical groups. For elaboration of the chemical name abbreviations see Supplemental Dataset.

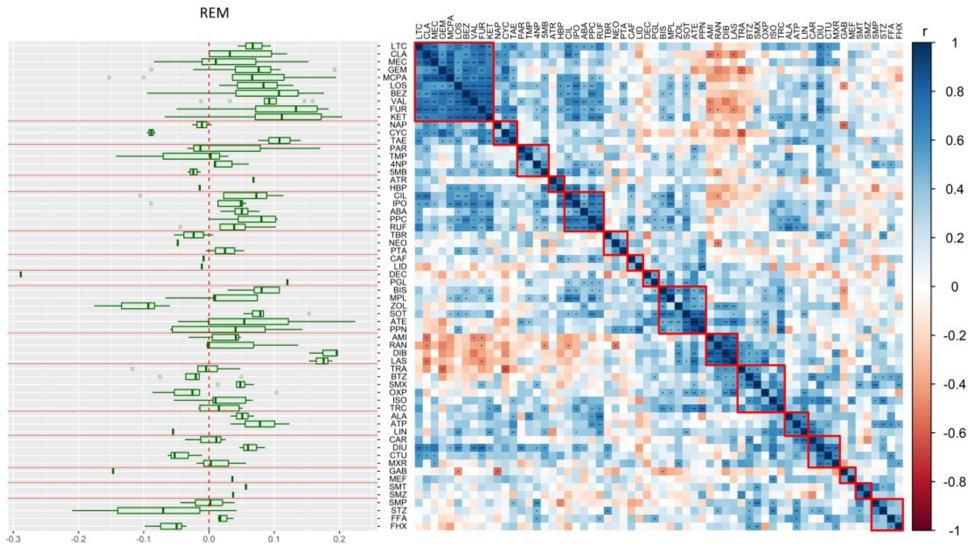

Figure S4. Hierarchical clustering heatmap (right) based on the magnitude of the Pearson correlation coefficient (r) of log k_{pH7} (d^{-1}). The significance of the correlations (P) is denoted by * ($P < 0.05$), ** ($P < 0.01$), *** ($P < 0.001$). The chemicals were classified into 16 clusters (chemical groups). In each group, the rate constants of all chemicals were benchmarked against one potential benchmark chemical. The boxplot (left) shows the magnitude of the reduction efficacy (REM), i.e., the change in the stddev of log k_{pH7} across systems for each chemical when it was used as the benchmark. Reduction in variance was a positive benchmark result. Red lines show the boundaries of the chemical groups. For elaboration of the chemical name abbreviations see Supplemental Dataset.

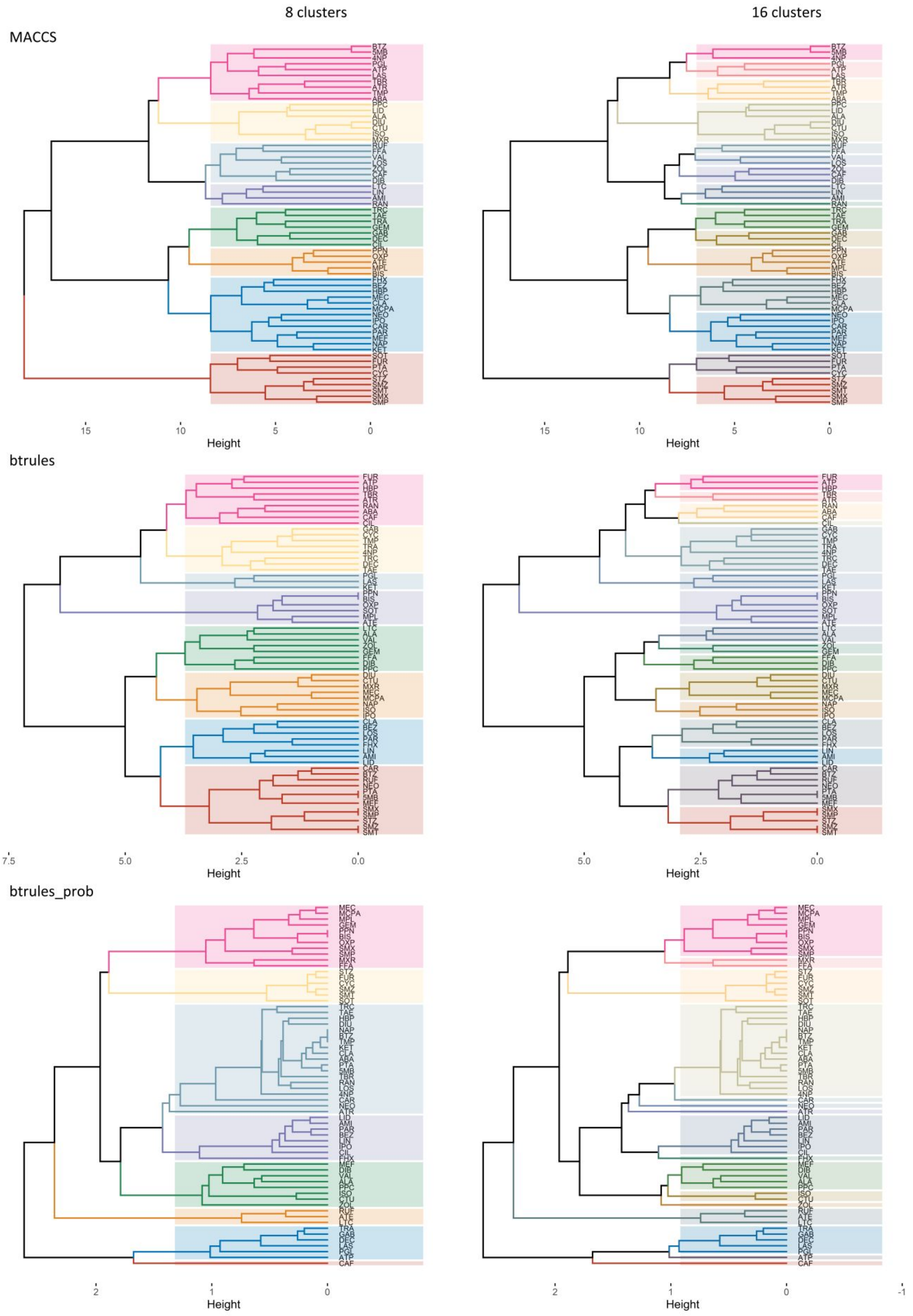

Figure S5. Agglomerative hierarchical clustering of chemicals based on MACCS (top), btrules (middle) and btrules_prob (bottom), showing dendrograms yielding eight clusters (left) and 16 clusters (right, marked by different colors). For elaboration of the chemical name abbreviations see Supplemental Dataset.

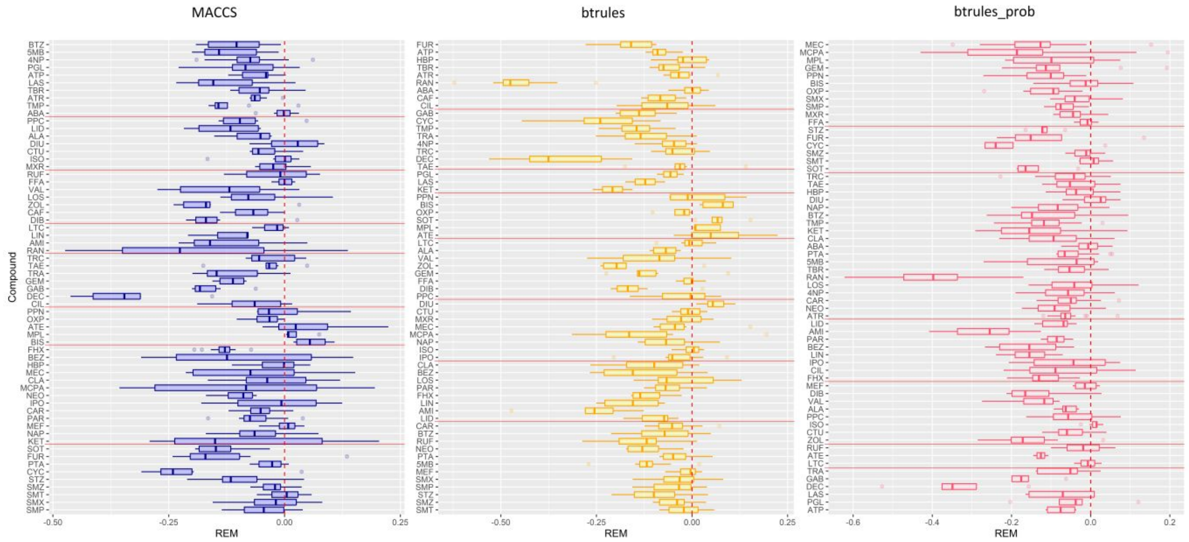

Figure S6. Boxplot showing the magnitude of the reduction efficacy (REM), i.e., the change in the stddev of log k_{pH7} across systems for each chemical within the group when the chemical on the y-axis was used as the benchmark for other chemicals in the same group. The grouping of chemicals was based on MACCS, btrules, and btrules_prob, respectively. Positive REM was a positive benchmark result. Red lines show the boundaries of the chemical groups. For elaboration of the chemical name abbreviations see Supplemental Dataset.
